# Quantitative measures of the vascular and neural components of the retina in adult individuals with congenital and untreated growth hormone deficiency

**DOI:** 10.1186/s40942-022-00408-x

**Published:** 2022-10-01

**Authors:** Nelmo V. Menezes, Cynthia S. Barros-Oliveira, Roberto Salvatori, Vinicius C. Gois, Cindi G. Marinho, Carla R. P. Oliveira, Viviane C. Campos, Alécia A. Oliveira-Santos, Hertz T. Santos-Júnior, Elenilde G. Santos, Enaldo V. Melo, Augusto C. N. Faro, Neima V. Oliveira, Hérika M. Gumes-Felix, Gustavo B. Melo, Manuel H. Aguiar-Oliveira

**Affiliations:** 1 Division of Ophthalmology, Health Sciences Graduate Program, Federal University of Sergipe, Aracaju, Sergipe, 49060–100 Brazil; 2 Division of Endocrinology, Health Sciences Graduate Program, Federal University of Sergipe, University Hospital, Street Claudio Batista s/n, Aracaju, SE 49060–100 Brazil; 3 Division of Endocrinology, Diabetes and Metabolism, Department of Medicine, The Johns Hopkins University School of Medicine, Baltimore, MD 21287 USA; 4 Retinal Specialist, Hospital de Olhos Rolemberg Gois, Aracaju, Sergipe, 49010-390 Brazil; 5 Hospital de Olhos de Sergipe, Aracaju, Sergipe, Brazil

**Keywords:** GH, GHRH receptor, IGF-I, Retina, OCT angiography

## Abstract

**Background:**

The somatotrophic axis, including hypothalamic growth hormone (GH)-releasing hormone (GHRH), pituitary GH and circulating IGF-I, is critical for body size. However, the local production of GH/IGF-I (and IGF-II) and other peptides is relevant for other body functions, such as vascular, brain, and retinal function. The consequences of GH deficiency (GHD) on the retinal structure are still unclear, possibly reflecting the heterogeneity of patients and the different types of assessment in previous publications. Our purpose was to assess quantitative measures of the vascular and neural components of the retina in subjects with severe congenital isolated GHD (IGHD).

**Methods:**

A cross-sectional study was carried out in 25 adult IGHD subjects and 25 age- and gender-matched controls. Interview, physical examination, laboratory data, optical coherence tomography (OCT) and OCT angiography (OCTA) were performed.

**Results:**

OCT revealed no difference in the areas of the nerve fiber layer average, nor in the areas of superior, inferior, or nasal quadrants, between the two groups. However, areas of the temporal quadrant (p = 0.041), the optical disc (p = 0.042), the cup (p < 0.0001), as well as the cup/disc ratio (p < 0.0001), were higher in IGHD subjects than controls. The rim area was smaller (p = 0.002), although still normal. In OCTA, there was no difference in the minimum foveal thickness, central fovea, foveal avascular zone, and retinal density in any assessed area.

**Conclusions:**

In conclusion, congenital IGHD does not affect quantitative measures of the vascular and neural retina, and it is associated with increased optical disc in this genetically homogeneous cohort.

## Introduction

The GH/Insulin-like growth factors (IGFs) system comprises the somatotrophic axis, critical for body size, including the hypothalamic GH-releasing hormone (GHRH), pituitary GH and circulating IGF-I, and the local production of GH/IGF-I, IGF-II [1], and other peptides like fibroblast growth factor (FGF), vascular endothelial growth factor (VEGF) and platelet-derived growth factor (PDGF), relevant for body functions, such as vascular, brain and ocular functions [2–4]. While vascular function is essential to guarantee blood supply to different tissues, the visual system is essential for neuromotor development, adaptation to the environment, and ultimately survival. Visual acuity depends on a well-developed eye, capable of generating images on the retina and processing them in the central nervous system [5]. The retina is composed by vascular and brain tissues, and both can be assessed non-invasively, by optical coherence tomography (OCT) and OCT angiography (OCTA) [6].

OCT provides measurement of the macula thickness, high-resolution cross-sectional images of the retina, optic nerve head and retinal nerve fiber layer thickness (RNFL), a measure of axonal and neuronal health in the anterior visual pathways [7]. Conversely, OCTA provides three-dimensional visualization of the perfused retinal and choroidal vasculature [8–10]. Unlike standard structural OCT, in addition to the intensity of the reflected light, OCTA also assesses the temporal changes of the OCT signal. Using repeated OCT section images (B-Scans) from the same location on the retina, temporal signal changes caused by moving particles (such as erythrocytes flowing through vessels) are detected, creating an image contrast between the perfused vessels and the surrounding static tissues. Through dense volume scans, it is possible to obtain OCTA images that are like fluorescence angiography images, which are the clinical gold standard, but require dye injection. In addition, while fluorescence angiography provides only two-dimensional images of the background, OCTA provides visualization of the structure and blood flow in the vitreous, retina and choroid, allowing to examine the different capillary networks of the retina, with vessel diameters around 8 μm [11].

Although historical studies suggest cardio or cerebrovascular vascular damage in acquired GH deficiency (GHD) [12, 13], more recent articles do not support this association, suggesting that vascular damage in acquired GHD may be linked to associated gonadal, thyroid, or cortisol deficits, their replacements and/or radiotherapy [14, 15]. We have described a large cohort of individuals residing in Itabaianinha, in the Brazilian state of Sergipe, with severe short stature, but near normal ocular axial length [16], due to a congenital isolated GHD (IGHD), caused by the c.57 + 1G > A mutation in the GH releasing hormone receptor (GHRHR) gene (*GHRHR* OMIM n.618157) [17]. These individuals otherwise normal pituitary function, and present extremely low serum GH levels throughout life and, in most cases, undetectable levels of serum IGF-I [18]. They also have visceral obesity [19], but with increased insulin sensitivity [20]. Despite high systolic blood pressure, increased levels of total and LDL cholesterol and C-reactive protein [21], they show no evidence of early atherosclerosis in the coronaries and aorta [22–24] and, consequently, have normal longevity [25]. Recently, we have also shown that cerebral vasoreactivity, a surrogate marker of cerebrovascular disease, is not impaired in these subjects [26]. Conversely, the consequences of GHD on the vascular and neural retinal aspects growth are still unclear, probably due to the acquired etiology of GHD in most previously published series, and the fact that GHD is often associated with additional pituitary deficits (thyrotrophic, corticotrophic and gonadotrophic hormones), whose replacement therapies are imperfect.

These IGHD subjects previously lived in the village of Carretéis and its rural surroundings in Itabaianinha city, an area surrounded by mountains, and subjected to high geographic isolation and, therefore, with a high frequency of consanguineous unions. The current increase in mobility of affected IGHD and of heterozygotes (reducing the birth of homozygous affected newborns), and the GH treatment of IGHD children (reducing the number of untreated IGHD adults make the in-depth study of this cohort even more important, as it will not last forever [27]. More than seventy articles have been published with this IGHD cohort, describing most aspects of the human organism, in a context of almost no pituitary GH secretion [27]. Consequently, this cohort is the only opportunity to study the interrelationships of the somatotrophic system with the retina in adults with untreated lifetime congenital IGHD, a cohort that cannot be found in other parts of the world.

We have previously analyzed the OCT of the macula and the fundus photography of these untreated IGHD adults. In comparison to local controls, they have a similar macular thickness assessed by OCT, and a moderate reduction in vascular branching points associated to an increased optic disc and cup size, assessed by a semi- quantitative method [5]. In the current study, we performed OCTA to generate volumetric scans at specific depths and, thus, obtain information about the structure and blood flow of the central retina and choroidal vasculature, and we analyzed the OCT of the optic nerve, to obtain quantitative measures of the vascular and neural components of the retina.

## Subjects and methods

### Subjects

In a cross-sectional study, IGHD and age and sex-matched normal stature control subjects were recruited by advertising placed on the Itabaianinha Dwarfs Association building, and by word of mouth among the inhabitants of the area. Inclusion criteria for IGHD were homozygosity for the c.57 + G > A *GHRHR* mutation, while homozygosity for the wild-type *GHRHR* allele was required for the control group. Exclusion criteria were age less than 20 years, previous GH replacement therapy, and impossibility of obtaining images due to severe cataracts, or mental problems. Estimating a medium effect of GH deficiency on the studied variables, and a power of 0.8 with α of 0.05, 20 to 30 individuals in each group would be needed. Of the currently living 54 IGHD individuals of this cohort [27], four are under 20 years of age, nine had previous treatment with GH, two had mental problems, and eight live distant from Itabaianinha, which reduced to 31 the total number of IGHD subjects suitable for the study, of which, twenty-six met our inclusion criteria One of them was excluded due to severe cataracts. We noted that there were three diabetics, with duration of diabetes of 7.0 (2.6) years [mean (standard deviation)]. From our database of genotyped homozygous normal subjects living in Itabaianinha, we recruited by phone calls and by word of mouth one control with the same age and sex for each IGHD subject. We also included three diabetics in the control group, with similar duration of diabetes to IGHD: 4.3 (4.9) years. Therefore, the IGHD group included 25 subjects with 10 women and 3 diabetics, range of age from 22 to 84 years. Similarly, the control group included 25 subjects with 10 women and 3 diabetics, range of age from 22 to 84 years.

### Interview, physical examination, and laboratory assessment

The subjects were first submitted to an interview including risk factors for cardiovascular disease such as hypertension, smoking, dyslipidemia, comorbidities, and their treatments. Subsequently, measurement of body weight, height, and blood pressure (average of three measurements after 10 min of rest in the sitting position with a cuff appropriate for the size of the arm) were carried out. Blood was collected after an overnight fast for glucose, hemoglobin A1C, total and HDL cholesterol, triglycerides, creatinine, and C-reactive protein (CRP), all measured by standard techniques, and LDL-C concentration calculated.

## Study protocol

### Optical coherence tomography (OCT)

Studies were performed without drug-induced mydriasis by using the Revo NX 130 device, Angio OCT-A with optical biometer Optopol Technology Sp. z o.o, 42–400, Zawiercie, Poland. We measured the nerve fiber layer; the thickness of four macular quadrants: superior, inferior, nasal, and temporal; the optic disc, the cup the rim area, a measure of the number of the ganglionic retinal ganglion cells, and the cup-to-disc ratio.

### Optical coherence tomography angiography (OCTA)

Quantitative measures of the superficial and deep capillary plexus were obtained by 6 × 6 mm scans. The size of the foveal avascular zone, the region surrounding the fovea, devoid of retinal capillaries and closely related to vision was obtained by 3 × 3 mm scans. The foveal avascular zone area and the perimeter were outlined manually along the innermost capillaries in the superficial capillary plexus. The software quantifies the vascular flow in the measured area and the retinal vessel density map was obtained by the percentage of flow in the inner circle (segments 1–5) of the grid of Early Treatment of Diabetic Retinopathy study (ETRDS), allowing evaluation of the central millimeter, the superior and inferior as well as the nasal and temporal ETRDS subfields [28, 29].

All images were digitally recorded and analyzed by the same experienced examiner (V.C.G.) blinded to the GH status. These images had a quality level of 10 (in a scale from 0 to 10), calculated by the device's own software. All these exams were performed at *Hospital dos Olhos Rolemberg Góis*, located in the city of Aracaju, the capital of Sergipe state, 120 km from Itabaianinha. The Federal University of Sergipe Institutional Review Board approved these studies, and all subjects gave written informed consent.

### Statistical analysis

Kolmogorov–Smirnov and Shapiro–Wilk test were used to test variables that have a normal distribution. The variables with normal distribution were expressed as mean (standard deviation) and compared by Student’s t test. The variables with non-normal distribution (glucose, hemoglobin A1C, the inferior quadrant, the cup area, the minimum foveal thickness, the foveal central sector, the superior retinal density, the temporal retinal density, the superior choroidal density, the inferior choroidal density, and the nasal choroidal density) were expressed as median (interquartile range) and compared by Mann–Whitney test. Categorical variables were compared by the Fisher’s exact test. We also provide the 95% confidence interval (95% CI) for each comparison of variables. For these analyses, the software IBM SPSS Statistics 23 (United States, 2015; RRID:SCR_002865) was used. Statistical significance was set at p < 0.05.

## Results

There was no significant difference in age [50 (15.1) vs. 50.5 (15.0) years, IGHD vs. control], sex, (15 men in each group), in the number of diabetes (three in each group) and on the number of smokers (two in the IGHD and none in the controls). Eight IGHD subjects had a history of hypertension (using eleven antihypertensive medications), while six in controls (using six antihypertensive medications); No statistical difference was present in the number of individuals with history of myocardial infarction (two in IGHD and none in controls), or stroke (none in both groups) between the groups. Similarly, there was no difference between the number of eyes with hypertensive retinopathy (two IGHD and five control eyes), and in the number of individuals with micro hemorrhages (none in IGHD eyes and four control eyes), or other retinal pathologies.

Table 1 shows the anthropometric measures, systolic blood pressure, diastolic blood pressure, and biochemical variables. Height, weight, and blood pressure were lower in the IGHD, but BMI and all biochemical variables were similar in the two groups, except for lower creatinine and higher C-reactive protein in the IGHD group.

**Table 1:** Comparison of anthropometric measures, systolic blood pressure (SBP), diastolic blood pressure (DBP), and biochemical variables between 25 IGHD and 25 controls. Data are expressed as mean (standard deviation), and compared by Student’s t test, except for glucose, hemoglobin A1C, expressed as median (interquartile range), and compared by Mann–Whitney test, and sex (male) expressed in n, (percentage)

Parameters	IGHD	Controls	95% CI	*p*
Age (years)	50 (15.1)	50.5 (15.0)	−8.6 to 8.5	0.993
Gender male (n, %)	15 (60%)	15 (60%)	−0.268 to 0.268	1.000
Height (m)	1.28 (0.1)	1.66 (0.1)	−0.4 to −0.3	<0.0001
Weight (Kg)	42.1 (10.7)	77.6 (12.13)	−42.3 to −28.9	<0.0001
BMI (Kg/m2)	25.7 (6.7)	28.1 (3.59)	−5.5 to 0.7	0.132
SBP (mmHg)	113.8 (18.9)	130.2 (15.9)	−28.5 to −4.5	0.008
DBP (mmHg)	72.9 (12.4)	83.0 (9.6)	−18.0 to −2.2	0.013
Fasting glucose (mg/dl)	87.0 (17.0)	81.9(20.5)	−19.2 to 10.5	0.369
Hemoglobin A1C (%)	5.7 (0.4)	5.4 (0.7)	−0.6 to 0.7	0.804
Total cholesterol (mg/dl)	218.0 (34.3)	221.8 (40.8)	−29.8 to 22.0	0.759
LDL-cholesterol (mg/dl)	147.1 (30.5)	146.7 (39.2)	−23.4 to 24.2	0.972
HDL-cholesterol (mg/dl)	44.1 (6.8)	45.2 (10.1)	−6.8 to 4.6	0.689
Triglycerides (mg/dl)	132.1 (64.4)	148.8 (60.9)	−15.6 to 21.9	0.480
Creatinine (mg/dl)	0.7 (0.1)	1.1 (0.3)	−0.5 to −0.28	<0.0001
C-reactive protein (mg/l)	6.1 (5.7)	2.8 (1.3)	0.5 to 6.0	0.022

The 95% confidence interval of difference (95% CI) is also showed

**Fig. 1:**
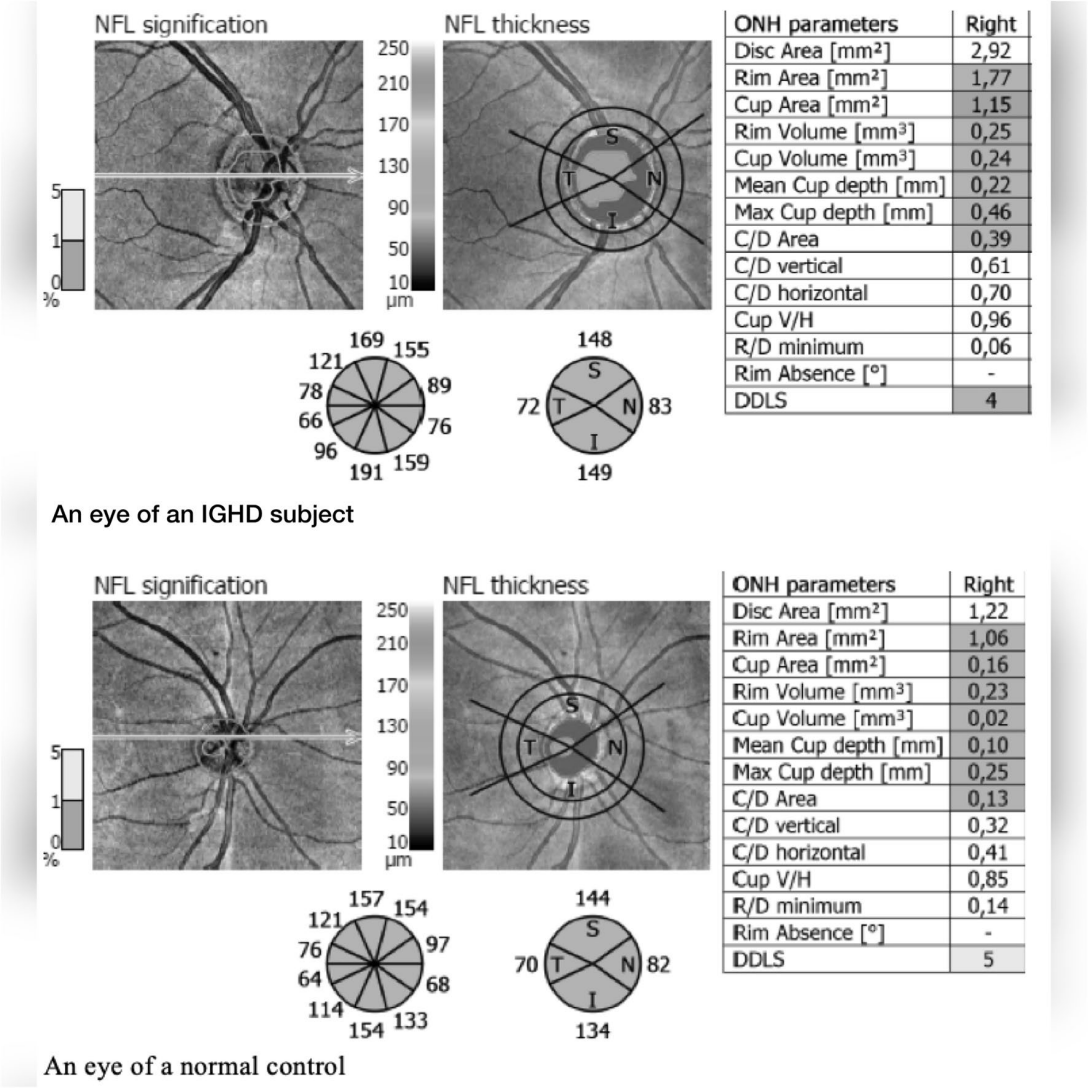
OCT of the optic nerve, with measurement of disc area, cup, rim, disc/rim ratio and ganglion cell layer measurement from an eye from a subject with IGHD, on the left, and from an eye from a normal control, on the right

Table 2 shows the macular OCT, and the optic nerve data of 50 IGHD and 50 controls eyes. No difference was found in the areas of nerve fiber layer average**,** nor in the areas of superior, inferior, or nasal quadrants. Areas of the temporal quadrant (p = 0.041), the optical disc (p = 0.042), the cup (p < 0.0001), as well as the cup/disc ratio (p < 0.0001) were higher in IGHD than controls. The rim area was smaller (p = 0.002) in IGHD, although within the normal range. Figure 1 shows the OCT of the optic nerve, with measurement of disc area, cup, rim, disc/rim ratio and ganglion cell layer from an eye from a subject with IGHD, on the left, and from an eye from a normal control, on the right.

**Table 2:** Comparison of the macula OCT and optic nerve data between 50 IGHD and 50 controls eyes. Data are expressed as mean (standard deviation), and compared by Student’s t test, except for the inferior quadrant and the cup area expressed as median (interquartile range) and compared by Mann–Whitney test

Parameters	IGHD	Controls	95% CI	*p*
Nerve fiber layer average (µm)	124 (12)	124 (15)	−7.43 to 2.98	0.399
Temporal quadrant (µm)	74 (12)	70 (8)	0.17 to 8.07	0.041
Nasal quadrant (µm)	100 (21)	104 (17)	−10.99 to 4.22	0.380
Superior quadrant (µm)	138 (19)	140 (15)	−8.84 to 4.68	0.543
Inferior quadrant (µm)	140 (19)	144 (19)	−11.41 to 2.30	0.190
Cup area (mm^2^)	0.97 (0.69)	0.44 (0.39)	0.24 to 0.61	<0.0001
Disc area (mm^2^)	2.22 (0.08)	2.02 (0.06)	0.01 to 0.40	0.042
Rim area (mm^2^)	1.23 (0.04)	1.45 (0.54)	−0.36 to −0.09	0.002
Cup/disc area ratio	0.42 (0.16)	0.27 (0.16)	0.09 to 0.22	<0.0001

The 95% confidence interval of difference (95% CI) is also showed

**Fig. 2:**
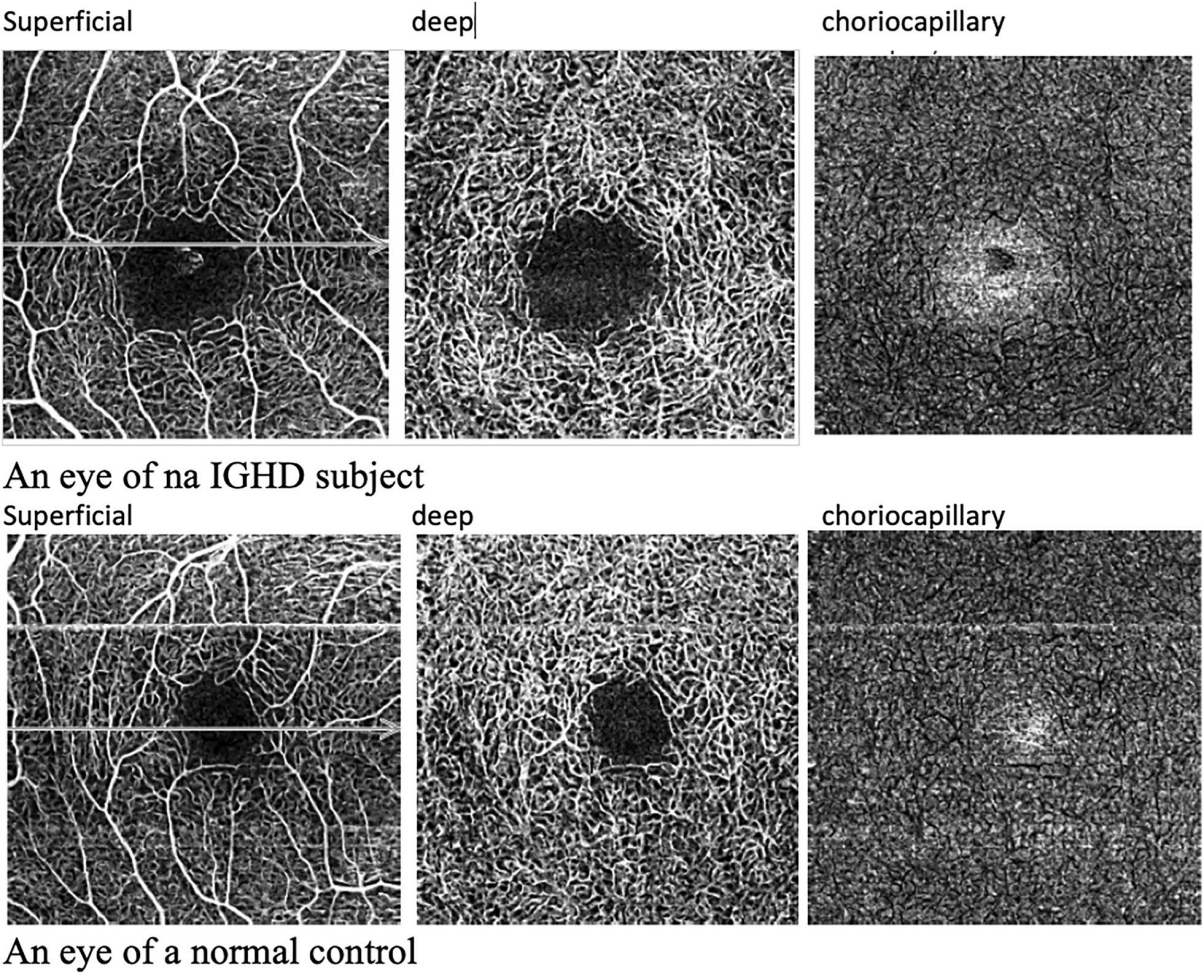
A map of vessel density in the superficial capillary and deep plexus, and choriocapillaris, from left to right, of an eye from a subject with IGHD above, and of an eye from a normal control below

Table 3 shows the OCTA data. There was no difference in minimum foveal thickness, central fovea, foveal avascular zone or retinal density in any area evaluated. Figure 2 shows a map of vessel density in the superficial capillary and deep plexus, and choriocapillaris from left to right of an eye from a subject with IGHD above, and of an eye from a normal control below.

**Table 3:** Comparison of the OCTA variables (minimum foveal thickness, the foveal central sector, foveal avascular zone, retinal and choroidal vascular density) between 50 IGHD and 50 controls eyes. Data are expressed as mean (standard deviation) for the foveal avascular zone, the inferior retinal density, the nasal retinal density, and the temporal choroidal density, and compared by Student’s t test The minimum foveal thickness, the foveal central sector, the superior retinal density, the temporal retinal density, the superior choroidal density, the inferior choroidal density, and the nasal choroidal density are expressed as median (interquartile range) and compared by Mann–Whitney test

Parameters	IGHD	Controls	95% CI	*p*
Minimum foveal thickness (µm)	179 (35)	184 (24)	−20.48 to 2.68	0.385
Foveal central sector (µm)	233 (33)	231 (34)	−16.40 to 3.68	0.576
Foveal avascular zone (µm^2)^	0.31(0.16)	0.35 (0.13)	−0.10 to 0.19	0.191
Superior retinal density (%)	11.4 (10)	11.9 (9.7)	−2.80 to 3.12	0.620
Inferior retinal density (%)	12.8 (8.7)	13.7 (6.8)	−3.03 to 3.16	0.964
Nasal retinal density (%)	16.8 (9.2)	17.2 (8.4)	−3.88 to 3.11	0.828
Temporal retinal density (%)	15.9 (7.3)	15.90 (11)	−3.82 to 2.70	0.617
Superior choroidal density (%)	3.9 (4.4)	7.6 (6.7)	−3.38 to 0.64	0.051
Inferior choroidal density (%)	7.7 (5.3)	9.7 (10)	−4.77 to 0.04	0.061
Nasal choroidal density (%)	10 (7.1)	9.7 (7.5)	−2.08 to 3.83	0.970
Temporal choroidal density (%)	13.2 (8.1)	13.4 (5.5)	−2.99 to 2.50	0.859

The 95% confidence interval of difference (95% CI) is also showed

## Discussion

The evolutive success of the human species depended on a body size able to obtain food and to reproduce, and on numerous body functions acting in an orchestrated manner. Among these, the functions of the sense organs, especially vision, are extremely important to survival. Visual acuity depends on a well-developed eye, capable of generating the images on the retina and processing them in the central nervous system [5]. The retina is a highly specialized structure, composed by brain and vascular tissues, assessed with increasing technical sophistication, from fundus photography, and fluorescein retinography to OCT and OCTA.

We have previously described a large cohort of IGHD individuals with severe short stature due to a homozygous inactivating mutation in the GHRHR gene [17], but near normal ocular axial length [16]. We have previously analyzed the OCT of the macula and the fundus photography of these untreated IGHD adults. In comparison to local controls, they had a similar macular thickness, and a moderate reduction in vascular branching points associated to an increased optic disc and cup size, assessed by a semi- quantitative analysis of fundus photography [5]. In the current paper, by using strictly quantitative methods, we confirm the increase in optical disc, the cup and in the cup-to-disc ratio. However, the moderate reduction in vascular branching points was associated with normal blood flow of the central retina and choroidal vasculature. We also show a simultaneous increase of the optic disc and cup size, with a reduction in the rim area. It is important to keep in mind that these differences may be statistically significant but not clinically significant, as still within normal values. However, this finding can guarantee preservation of enough retinal ganglion cell number, whose survival is influenced by local GH, as both GH and GH receptors were demonstrated in these cells [30]. Our data raised in adults, corroborate the hypothesis that an increased optic disc could predict the possibility of GHD in a child with severe short stature [5, 31]. Our IGHD individuals are part of a unique large pedigree and therefore are subject to other genetic influences, in addition to the inactivating *GHRHR* OMIM n.618157 mutation, which in homozygosity causes IGHD. Although it is well known that the optic disc and the cup to disc relationship have high heritability and familial aggregation [32], we assume that the increase in these structures must be incorporated into the phenotypic characteristics of this homozygous mutation. Interestingly, IGF-I treatment in individuals with Laron Syndrome, a model of GH unresponsiveness due to mutations in the GH receptor gene, seems to reduce the measures of the optic disc and cup [33].

Another novel finding of the present study is the normal nerve fiber layer thickness in these subjects, coinciding with the normality of the macular thickness. Nerve fiber layer thickness was reported as thinner in congenital (not proven genetically) treated GHD children [34], in children with acquired GH deficiency [35], and in adult women with Sheehan syndrome, a model of acquired hypopituitarism with GH deficiency [36]. GH-producing cells can be identified in the retina at nine weeks of gestation, while embryonic neural development occurs at an earlier period [37]. The normality of the normal nerve fiber layer thickness in our subjects argues against a possible role of pituitary GH and circulating IGF-I on the neural development of the retina as previously suggested [35]. It is commonly accepted that IGF-II has a greater effect when fetal somatic and ocular development is considered. We do not know if this peptide has an effect in the neural development of the retina in IGHD, as IGF-II is upregulated in these subjects [18].

Another finding of the present study is that all the measures of OCTA retinal vessel architecture were similar between IGHD subjects and controls, suggesting that the somatotrophic axis is not critical for the development of retinal vessels, at least in a micrometric scale. In these IGHD individuals, there appears to be a difference between the behavior of retinal vessels visible on fundus photography, showing fewer branching points, and retinal vascular density as assessed by OCTA. The retinal morphology at fundoscopy parallels with the benign behavior of their carotid intimal medial thickness, which remains similar to the control in these IGHD subjects after 14 years of follow-up [22, 26]. On other hand, their normal OCTA retinal vessel micro-architecture seems to parallel the normal behavior of their cerebral vasoreactivity, measured by the transcranial Doppler, a surrogate marker of cerebrovascular disease [26]. The normality of the minimum foveal thickness, the central fovea, the foveal avascular zone, and the retinal and choroidal vascular density in IGHD subjects, suggests the presence of local compensatory mechanisms, guaranteeing their normality of the microvascular retinal architecture. Local compensatory mechanisms are also involved in the teeth, ocular, and brain growth [38]. Together, teeth, eye, brain, and retinal development may involve different patterns of regulation than the whole-body growth, suggesting in these sites, other regulatory mechanisms beside the somatotrophic axis.

A strength of this work is the strict matching process between the two groups. Only blood pressure and creatinine were lower, and CRP was higher in the IGHD group. The lower blood pressure in IGHD likely reflects the antihypertensive treatment offered to these individuals, who are known to have a slight increase in blood pressure [21]. Lower creatinine levels reflect their smaller muscle mass [23]. Higher CRP is a well-known feature of this group [21], reflecting an inverse mutual reaction between CRP and GH. Indeed, CRP is significantly reduced in acromegaly, a state of GH hypersecretion [39]. An additional merit of this work is its interdisciplinary approach, harmonizing the links between ophthalmology, endocrinology, and genetics. The relatively low number of subjects may appear as a limitation of this work. However, considering the rarity of IGHD, and the fact that GHD is treated in most parts of the world, this is actually a uniquely large group.

In conclusion, congenital IGHD does not affect quantitative measures of the vascular and neural retina, and it is associated with increased optical disc in this genetically homogeneous cohort.

## Data Availability

The datasets used and/or analyzed during the current study are available from the corresponding author on reasonable request.

## References

[1] Aguiar-Oliveira MH, Bartke A (2019). Growth hormone deficiency: health and longevity. Endocr Rev.

[2] Delafountaine P, Song YH, Li Y (2004). Expression, regulation, and function of IGF-1, IGF-1R and IGF-1 binding proteins in blood vessels. Arterioscler Thromb Vasc Biol.

[3] Caicedo D, Díaz O, Devesa P, Devesa J (2018). Growth hormone (GH) and cardiovascular system. Int J Mol Sci.

[4] Harvey S, Martinez-Moreno CG (2016). Growth hormone and ocular dysfunction: endocrine, paracrine, or autocrine etiologies?. Growth Horm IGF Res.

[5] Pereira-Gurgel VM, Faro ACN, Salvatori R, Chagas TA, Carvalho-Junior JF, Oliveira CRP (2016). Abnormal vascular and neural retinal morphology in congenital lifetime isolated growth hormone deficiency. Growth Horm IGF Res.

[6] Selvam S, Kumar T, Fruttiger M (2018). Retinal vasculature development in health and disease. Prog Retin Eye Res.

[7] Sakata LM, DeLeon-Ortega J, Sakata V, Girkin CA (2009). Optical coherence tomography of the retina and optic nerve-a review. Clin Ex Ophthalmol.

[8] Matsunaga D, Yi J, Puliafito CA, Kashani AM (2014). OCT angiography in healthy human subjects. Ophthalmic Surg Las Imag Retin.

[9] Spaide RF, Klancnik JM, Cooney MJ (2015). Retinal vascular layers imaged by fluorescein angiography and optical coherence tomography angiography. JAMA Ophthalmol.

[10] de Carlo TE, Romano A, Waheed NK, Duker JS (2015). A review of optical coherence tomography angiography (OCTA). Int J Retina Vitreous.

[11] Tan PEZ, Yu PK, Balaratnasingam C, Cringle SJ, Morgan WH, McAllister IL (2012). Quantitative confocal imaging of the retinal microvasculature in the human retina. Invest Oftalmol Vis Sci.

[12] Rosen T, Bengtsson BA (1999). Premature mortality due to cardiovascular disease in hypopituitarism. Lancet.

[13] Bülow B, Hagmar L, Mikoczy Z, Nordström C-H, Erfurth EM (1997). Increased cerebrovascular mortality in patients with hypopituitarism. Clin Endocrinol.

[14] Burman P, Mattsson AF, Johannsson G, Höybye C, Holmer H, Dahlqvist P (2013). Deaths among adult patients with hypopituitarism: hypocortisolism during acute stress, and de novo malignant brain tumors contribute to an increased mortality. J Clin Endocrinol Metab.

[15] Biscotto IP, Hong VAC, Batista RL, Mendonca BB, Arnhold IJP, Bortolotto LA (2021). Vasculometabolic effects in patients with congenital growth hormone deficiency with and without GH replacement therapy during adulthood. Pituitary.

[16] Faro ACN, Pereira-Gurgel VM, Salvatori R, Campos VC, Melo GB, Oliveira FT (2017). Ocular findings in adult subjects with an inactivating mutation in GH releasing hormone receptor gene. Growth Horm IGF Res.

[17] Salvatori R, Hayashida CY, Aguiar-Oliveira MH, Phillips JA, Souza AHO, Gondo RG (1999). Familial dwarfism due to a novel mutation of the growth hormone-releasing hormone receptor gene. J Clin Endocrinol Metab.

[18] Aguiar-Oliveira MH, Gill MS, Sde E, Barretto A, Alcântara MRS, Miraki-Moud F (1999). Effect of severe growth hormone (GH) deficiency due to a mutation in the GH-releasing hormone receptor on insulin-like Growth Factors (IGFs), IGF-binding proteins, and ternary complex formation throughout life. J Clin Endocrinol Metab.

[19] Gomes-Santos E, Salvatori R, Ferrão TO, Oliveira CRP, Diniz RDCA, Santana JAM (2014). Increased visceral adiposity and cortisol to cortisone ratio in adults with congenital lifetime isolated GH deficiency. J Clin Endocrinol Metab.

[20] Oliveira CR, Salvatori R, Barreto-Filho JA, Rocha IES, Mari A, Pereira RMC (2012). Insulin sensitivity and β-cell function in adults with lifetime, untreated isolated growth hormone deficiency. J Clin Endocrinol Metab.

[21] Barreto-Filho JA, Alcantara MR, Salvatori R, Barreto MA, Sousa ACS, Bastos V (2002). Familial isolated growth hormone deficiency is associated with increased systolic blood pressure, central obesity, and dyslipidemia. J Clin Endocrinol Metab.

[22] Oliveira JLM, Marques-Santos C, Barreto-Filho JA, Filho RX, Britto AVO, Souza AHO (2006). Lack of evidence of premature atherosclerosis in untreated severe isolated growth hormone (GH) deficiency due to a GH-releasing hormone receptor mutation. J Clin Endocrinol Metab.

[23] Costa UMM, Oliveira CRP, Salvatori R, Barreto-Filho JAS, Campos VC, Oliveira FT (2016). Brazilian adult individuals with untreated isolated GH deficiency do not have accelerated subclinical atherosclerosis. Endocr Connect.

[24] Souza AHO, Farias MIT, Salvatori R, Silva GMF, Santana JAM, Pereira FA (2014). Lifetime, untreated isolated GH deficiency due to a GH-releasing hormone receptor mutation has beneficial consequences on bone status in older individuals and does not influence their abdominal aorta calcification. Endocrine.

[25] Aguiar-Oliveira MH, Oliveira FT, Pereira RMC, Oliveira CRP, Blackford A, Valenca EHO (2010). Longevity in untreated congenital growth hormone deficiency due to a homozygous mutation in the GHRH receptor gene. J Clin Endocrinol Metab.

[26] Marinho CG, Melo HA, Salvatori R, Nunes MAP, Oliveira CRP, Campos VC (2020). Cerebral vasoreactivity, a surrogate marker of cerebrovascular disease, is not impaired in subjects with lifetime, untreated, congenital isolated GH deficiency. Endocrine.

[27] Aguiar-Oliveira MH, Salvatori R (2021). Disruption of the GHRH receptor and its impact on children and adults: the Itabaianinha syndrome. Rev Endocr Metab Disord.

[28] Rezar-Dreindl S, Eibenberger K, Told R, Neumayer T, Steiner I, Sacu S (2021). Retinal vessel architecture in retinopathy of prematurity and healthy controls using swept-source optical coherence tomography angiography. Acta Ophthalmol.

[29] Saif PS, Salman AERG, Omran NAH, Farweez YAT (2020). Assessment of diabetic retinopathy vascular density maps. Clin Ophthalmol.

[30] Sanders EJ, Parker E, Harvey S (2009). Endogenous growth hormone in human retinal ganglion cells correlates with cell survival. Mol Vis.

[31] Collett-Solberg PF, Liu GT, Satin-Smith M, Katz LL, Moshang T (1998). Pseudopapilledema and congenital disc anomalies in growth hormone deficiency. J Pediatr Endocrinol Metab.

[32] Klein BE, Klein R, Lee KE (2004). Heritability of risk factors for primary open-angle glaucoma: the beaver dam eye study. Invest Ophthalmol Vis Sci.

[33] Bourla DH, Weinberger D, Laron Z, Kopchick JJ (2011). Ocular findings in laron syndrome. Laron syndrome from man to mouse: lessons from clinical and experimental experience.

[34] Nalcacioglu-Yuksekkaya P, Sen E, Yilmaz S, Elgin U, Gunaydin S, Aycan Z (2014). Decreased retinal nerve fiber layer thickness in patients with congenital isolated growth hormone deficiency. Eur J Ophthalmol.

[35] Yüce Ö, Yalçın NG, Bideci A, Döğer E, Emeksiz HC, Hasanreisoğlu M (2018). Retinal neural and vascular structure in isolated growth hormone deficiency children and evaluation of growth hormone treatment effect. J Clin Res Pediatr Endocrinol.

[36] Atmaca M, Kızıldağ E, Candan Z, Özbay MF, Seven İ (2015). Ocular findings in Sheehan's syndrome. Graefes Arch Clin Exp Ophthalmol.

[37] Sperling M (2002). Disorders of growth hormone/insulin-like growth factor secretion and action. Pediatr Endocrinol.

[38] Oliveira Neto LA, Nascimento JKF, Salvatori R, Oliveira-Santos AA, Girão RS, Silva EV (2022). Growth of teeth and bones in adult subjects with congenital untreated isolated growth hormone deficiency. Growth Horm IGF Res.

[39] Verhelst J, Velkeniers B, Maiter D, Haentjens P, T’Sjoen G, Rietzschel E (2013). Active acromegaly is associated with decreased hs-CRP and NT-proBNP serum levels: insights from the Belgian registry of acromegaly. Eur J Endocrinol.

